# Examining the Role of Duration and Frequency of Homelessness on Health Outcomes Among Unsheltered Young Adults

**DOI:** 10.1016/j.jadohealth.2023.06.013

**Published:** 2023-08-12

**Authors:** Jessica Richards, Benjamin F. Henwood, Natalie Porter, Randall Kuhn

**Affiliations:** a Department of Community Health Sciences, Jonathan and Karin Fielding School of Public Health, University of California Los Angeles, Los Angeles, California; b Suzanne Dworak-Peck School of Social Work, University of Southern California, Los Angeles, California

**Keywords:** Homelessness, Young adults, Health, Weathering, Chronic, Episodic

## Abstract

**Purpose:**

We examined the impact of duration and number of homelessness episodes on health outcomes for unsheltered homeless young adults.

**Methods:**

We analyzed the 2018/2019 Los Angeles County homeless youth demographic surveys. We addressed five summary health outcomes: physical health, mental health, substance use disorder, tri-morbidity, and any condition. Respondents were classified into three homeless trajectory groups: (1) short-term–homeless < 1 year in one episode, (2) episodic–homeless < 1 year and multiple episodes, and (3) long-term–homeless continuously for > 1 year. Weighted bivariate and multivariate logistic regression models tested the relationship between homeless trajectory group and health, with controls for sociodemographic factors and structural exposures.

**Results:**

Mental health and substance use were high among unsheltered young adults compared to national rates. Long-term homeless respondents were significantly more likely than short-term to report a mental health condition (53.3% vs. 39.8%, *p* < .001), substance use disorder (25.5% vs. 18.3%, *p* < .001), and physical conditions (26.0% vs. 15.6%, *p* = .008). Episodic respondents were more likely to report a mental health condition (50.5%, *p* < .001). In multivariate models, long-term respondents had twice the odds of tri-morbidity (odds ratio [OR] = 2.14, *p* < .05) and any health condition (OR 2.00, *p* < .01) as short term and significantly higher odds of a physical health condition (OR = 1.64, *p* < .05).

**Discussion:**

Youth with longer durations and more frequent episodes of homelessness have substantially poorer health outcomes. The association of longer duration to poorer health persisted in multivariate models. Longer duration of unsheltered homelessness may drive the onset of physical and mental health problems.

It is estimated that one in 10 young adults (aged 18–24 years) experience homelessness in the United States each year [1]. Compared to their housed counterparts, these young adults report higher rates of risky sexual behaviors (including trading sex for shelter), substance use, and poor physical and mental health [2–6]. Within this population, evidence suggests that longer durations of homelessness and more frequent episodes of homelessness may contribute to worse health outcomes [7–9].For example, studies have found that compared to newly homeless young adults, those homeless for > 6 months were more likely to use drugs intravenously, engage in sex work, have at least four sexual partners, and report worse physical and mental health [10,11]. A study of homeless adolescents found that for each additional year of age, there was a 37% reduction in the likelihood of exiting [10]. In addition to duration, research on adults highlights the unique burdens facing those with multiple episodes of homelessness, whose “episodic” experiences may be associated with destabilizing transitions between homelessness, carceral systems, institutions, or unsafe residential arrangements [12]. Furthermore, the chances of exiting homelessness in later life may decrease with longer duration and more frequent episodes of young adult homelessness [13,14].

The purpose of this study is to examine the impact of duration and number of homelessness episodes on health outcomes for unsheltered homeless young adults, which refers to those living on the street, in cars, abandoned buildings, and other places not meant for human habitation. Research suggests that unsheltered homelessness versus sheltered homelessness contributes to worse health outcomes [8,15], yet few studies have specifically focused on unsheltered young adults. Our lack of understanding of health risks facing unsheltered young adults limits our ability to address health needs and set priorities for homelessness prevention and early intervention. Furthermore, the relationship between health and homeless-ness in young adults experiencing unsheltered homelessness is not fully understood; in particular, whether increased duration and frequency of homelessness leads to worse health outcomes, or whether youth with health challenges are at an increased risk of becoming homeless.

While we hypothesize that rates of physical health conditions, mental health conditions, and substance use disorder will be highest among unsheltered young adults with longer durations and more frequent episodes of homelessness, for this study, duration and frequency of homelessness were combined and operationalized as homeless trajectory groups based on previous work demonstrating differential experiences and health outcomes of homelessness by trajectory [16–18]. Specifically, unsheltered young adults were classified into three homeless trajectory groups: (1) short-term–homeless less than a year and one time, (2) episodic–homeless less than one year and more than one time, and (3) long-term–homeless more than or equal to one year. While we expect that rates of physical health conditions, mental health conditions, and substance use disorder will be highest among unsheltered youth experiencing episodic and long-term homelessness, we further test whether these differences can be explained by observable differences in the sociodemographic background and structural burdens characterizing individual case histories, or whether differences by trajectory persist even in the presence of statistical controls for case history.

## Methods

### Study overview

This study leverages existing deidentified data collected as part of the Los Angeles Homeless Services Authority (LAHSA) annual homeless count. In 2020, there were more than 4,100 youth who were homeless in Los Angeles County, which is the largest population of homeless youth in the United States and a 19% increase from 2019 [19–21]; more than half (58%) were unsheltered. For this study, we conducted secondary data analysis using the LAHSA youth survey data from 2018 to 2019 for unsheltered young adults (aged 18–24 years; 2020 data were omitted due to changes to question wording). Weighted averages were used to adjust for survey design and make estimates representative of Los Angeles County’s unsheltered youth population. Sample characteristics are included, and multivariate logistic regression models were used to test the relationship between homeless trajectory groups and summary health outcome measures. The study was reviewed by the University of California, Los Angeles Institutional Review Board (IRB#21–000162), which determined that the study was exempt from human subjects review.

### Data source and sampling

As part of its annual federally mandated homeless count, LAHSA conducts an enumeration and survey of unsheltered young adults aged 18–24 years that covers the Los Angeles Continuum of Care geographic area [22]. A stratified random sampling method was used for the youth survey [22,23]. First, census tracts were stratified by geographic region and whether the tract was a youth/young adult hotspot. Hotspot strata were defined based on historical data and expert/provider estimates to target areas where homeless youth congregate. Census tracts were then sampled using Neyman proportional allocation methods based on the average number of individuals per census tract. To avoiding undercounting hidden subpopulations, youth in sampled tracts were identified in two ways: (1) they were surveyed by street teams deployed in selected census tracts and (2) they were surveyed by youth homelessness providers at community organizations that served as designated survey sites [22]. Young adults aged 18–24 years were eligible to participate if they had stayed in an unsheltered location most of the last 30 days (stayed in an unsheltered location last night was added to the 2019 survey).

### Measures

#### Homelessness.

Homeless trajectory categories were developed based on the length of one’s current period of homelessness and total number of episodes of homelessness in the past three years. Homelessness was measured using two items: (1) “How long have you been experiencing homelessness this time?” and (2) “In the past three years, what about the number of separate times you experienced homelessness, on the street, in a vehicle, or in shelters?” Current duration was converted to years homeless to facilitate analysis and data interpretation. Homeless durations equal to or more than one year have been used to operationalize long-term homelessness. Survey respondents indicated the number of homeless episodes in the past three years: one time, 2–3 times, or four or more times. Duration and episode categories were combined to produce three homeless trajectory groups: (1) short-term–homeless less than a year and one time (n = 473), (2) episodic–homeless less than 1 year and more than one time (n =356), and (3) long-term–homeless more than or equal to one year (n = 807). Reason for homelessness was assessed using to choose all that apply question “what do you think are some of the main reasons or conditions that led to your loss of housing?”

#### Health.

Outcome variables include 10 health conditions which were collapsed into five summary health outcomes: physical health condition, mental health condition, substance use disorder, tri-morbidity, and any health condition. Health conditions were assessed using the question “do you have, have you ever had, or has a healthcare provider ever told you that you have any of the following health conditions?” Youth who reported a physical disability, developmental disability, physical illness, human immunodeficiency virus, or brain injury were identified as having a physical health condition. Those who reported severe depression, serious and long continuing mental illness, or post-traumatic stress disorder were identified as having a mental health condition. Those who reported problematic alcohol use or problematic drug use were identified as having a substance use disorder. Disabling condition was not used in conjunction with health outcomes due to an inability to identify which health condition was disabling. Tri-morbidity was defined as having a physical health condition, mental health condition, and substance use disorder. Having any health condition was defined as having a physical health condition, mental health condition, or substance use disorder.

#### Covariates.

Demographic characteristics and structural risk factors for homelessness were included as covariates. Age, gender, sexual orientation, race, ethnicity, and veteran status have been associated with homelessness, chronic homelessness, or both. Transgender and gender nonconforming categories were combined for gender but not for sexual orientation. Although transgender people may identify as a sexual minority (i.e., LGBQ), researchers recommend including transgender young adults as a distinct category to avoid conflating gender identity-related experiences with minority sexual orientation [24]. Due to relatively small sample sizes, Asian (n = 13), American Indian or Alaska Native (n = 18), Native Hawaiian or Pacific Islander (n = 8), and multiple race respondents (n = 60) were combined with other race (n = 44). Race and ethnicity were combined into race/ethnicity (we use “White” to refer to non-Hispanic Whites and “Black” to refer to non-Hispanic Black/African Americans) where ethnicity supersedes race. For example, a positive response to Hispanic is reported as Hispanic regardless of race.

Structural risk factors for homelessness were measured using the following variables: history of domestic violence, highest level of education completed, employment status, involvement with the justice system, involvement with the child welfare system, and receipt of government assistance. Young adults who reported physical abuse, sexual abuse, stalking, or dating violence were identified as having experienced domestic violence. Involvement with the justice system was defined as having selected “yes” to being involved in any of the following justice systems: juvenile detention or probation camp, juvenile probation group home/residential program, juvenile home probation, jail, prison, adult probation, or parole. Involvement with the child welfare system was defined as having selected “yes” to being involved in any of the following child welfare systems: foster care placement with extended family or nonrelative family, foster care residential or group home placement, extended foster care (AB-12), independent living program, or supervised independent living program (2019 only).

### Analysis

Analysis was conducted using Stata 16.0. Multivariate weighted logistic regression models were used to test the relationship between homeless trajectory groups and summary health outcome measures. Sample weights were constructed for use with youth survey data and calculated by taking the inverse of the probability of census tract selection within each stratum. Interviewer-perceived characteristics (e.g., age, gender, race, ethnicity) were collected for all potential respondents and were used to adjust for nonresponse (unapproachable, refused or declined, or did not provide eligibility information) in final survey weights. Survey year was included in regression models to control for variation by year and geographic location. For multivariate analysis, responses of “not reported” (3.4% of total) were combined with responses for “yes” for domestic violence. This was done to reduce multicollinearity and because bivariate coefficient estimates were highly similar for individuals with “yes” or “not reported” status. To reduce multicollinearity, a small number of young adults who were disabled or on disability (3.4%) were combined with unemployed young adults for employment status.

## Results

### Sociodemographic characteristics

The sample included 1,672 unsheltered young adults, with 1,220 included in the final analytic sample for multivariate analysis. Data were missing for frequency of homeless episodes (n = 36) and health conditions (n = 114); the remainder of young adults were missing at least one covariate (n = 302). Given the high level of missingness in the data, we first tested for differences between individuals with complete data who were included in the analysis versus those who were excluded. Sociodemographic characteristics did not differ significantly between included and excluded youth except by gender, with a greater proportion of excluded young adults identified as transgender or gender nonconforming.

Sociodemographic characteristics by homeless trajectory are summarized in the top half of Table 1. The mean age of study participants was 22.6 years (standard deviation = 1.9). There were more males (64.4%) than females (31.7%) with a minority identifying as transgender or gender nonconforming (1%). Although most of the sample identified as heterosexual (78.3%), 21.7% identified as gay or lesbian, bisexual, or unsure/questioning (LGBQ). This finding is consistent with previous research that estimates between 20% and 40% of homeless youth identify as LGBTQ and much higher than the estimated 4.6% of adults who identify as LGBT in Los Angeles [24,25]. Most unsheltered youth were people of color: Hispanic (41.1%), Black (27.5%), and other races (9.6%) compared to 21.9% of White young adults. Among this unsheltered homeless sample, key race/ethnic groups were over-represented relative to their share of the LA County age 20–24 population, including Black (27.5% vs. 7.6% general population), American Indian/Alaska Native (3.7% vs. 0.2%), and multiracial (9.9% vs. 2.6%) [26]. The majority were unemployed (75.9%), although 18% reported being employed either through full-time or part-time work, seasonal/temporary work, or being self-employed. Demographic characteristics were similar across trajectory groups, although youth experiencing episodic or long-term homelessness were less likely to be female, Hispanic or Black, and Bisexual or unsure/questioning.

A large proportion of youth (43.5%) reported domestic violence, with significantly higher experience of domestic violence among those with episodic trajectory (53.6%) than those with long-term (43.5%) or short-term (37.5%). Most respondents had a high-school degree or higher (61.1%). More than half reported involvement in criminal justice systems (58.7%) and a third reported involvement in child welfare systems (33.5%). Levels of justice involvement were significantly higher among long-term (68.1%) and episodic (54.5%) than among short-term (46.6%). Levels of child welfare involvement were also higher (36.9% long-term, 41.5% episodic, and 23.2% short-term). Among the 66.3% of youth who received government assistance, the most common assistance programs were Medicaid/MediCal/LA Care (37.3%), food stamps/EBT/CalFresh (28.4%), and general relief/general assistance (25.2%).

Health conditions are summarized in Table 2. Rates of self-reported health and substance use were high among unsheltered young adults compared to national rates (aged 18–25 years). Nearly half of unsheltered young adults reported having a mental health condition (48.5%) compared to 29.4% in the United States generally [27]. Almost a quarter reported a substance use disorder (22.4%) compared to 14% of young adults nationally [28]. The prevalence of comorbid mental illness and substance use disorder was 16.1%. Unsheltered young adults were more likely to report problematic drug use (19%) than alcohol use (11.1%). Rates of self-reported physical health conditions (20.9%) were lower than mental health conditions. Significant differences by homeless trajectory were observed in each of the three individual mental health items, resulting in highly significant differences in any mental health condition (53.3% long-term, 50.5% episodic, and 39.8% short-term; *p* < .001 in pairwise tests). Long-term respondents were significantly more likely than short-term to report problematic drug use (21.9% vs. 15.3%, *p* < .001) and any substance use disorder (25.5% vs. 18.3%, *p* < .001), while episodic were moderately more likely to report any substance use disorder (21.3% vs. 18.3%, *p* = .021). Few significant differences were found for individual physical health condition, but long-term were significantly more likely than short-term to report at least one physical health condition (26.0% vs. 15.6%, *p* = .008). As a result, there were also significantly higher reports of any health condition and tri-morbidity among long-term and episodic respondents than short-term.

**Figure 1.**
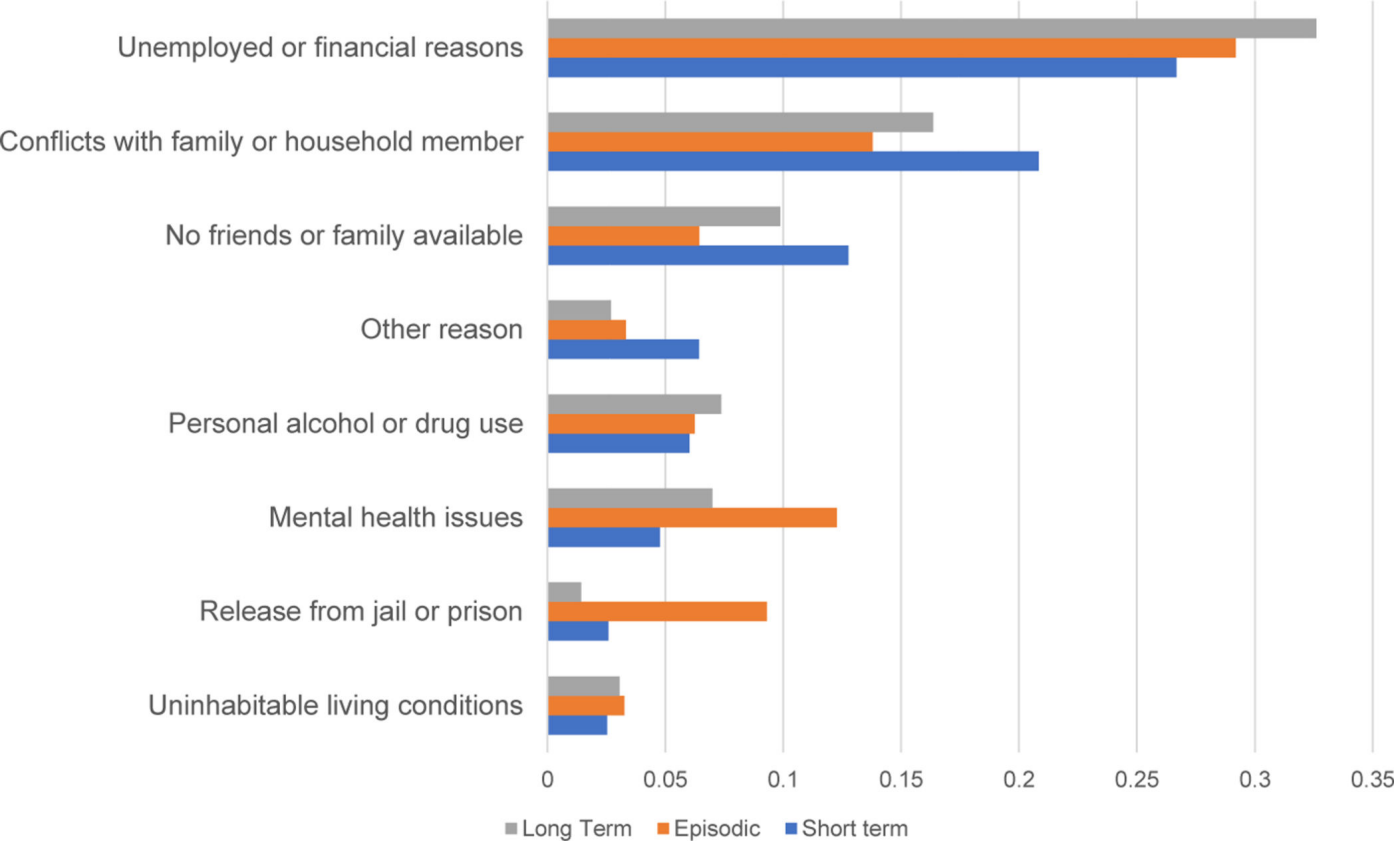
Reason for homelessness by homelessness trajectory group.

The primary reasons unsheltered youth reported for homelessness are presented in Figure 1. The most common reasons included unemployed or financial reasons (30.1%), conflicts with family or household members (17.1%), no friends or family available (10.0%), mental health issues (7.4%), and personal alcohol or drug use (6.7%). The top two reasons for homelessness were consistent across homeless trajectories. Those experiencing episodic homelessness were distinct in reporting mental health issues (12.3%) and release from jail or prison (9.3%) as more common reasons than other groups.

#### Multivariate models

Multivariate logistic regression was used to model the odds of having a physical health condition, mental health condition, substance use disorder, all three conditions (tri-morbidity), and any health condition (Table 3). Even after controlling for sociodemographic and structural risk factors, young adults experiencing long-term homelessness had double the odds of tri-morbidity (odds ratio [OR] = 2.14, *p* < .05) and having any health condition (OR 2.00, *p* < .01) compared to short-term.

Among individual health measures, long duration remained significantly associated with poor physical health (OR = 1.64, *p* < .05) but not mental health or substance abuse. Differences between episodic and short-term were not significant for any health measure, indicating that bivariate relationships were explained by significant relationships between control measures and health outcomes.

We highlight key results pertaining to the sociodemographic controls. Being female was associated with lower odds of having a physical health condition (OR = 0.52, *p* < .05), substance use disorder (OR = 0.49, *p* < .01), and any health condition (OR = 0.56, *p* < .01) compared to males. Transgender and gender nonconforming respondents did not differ from male respondents on physical health, mental health, or substance use outcomes but were significantly more likely to have all three conditions (OR = 2.75, *p* < .01). LGBQ youth had greater odds of having a physical health condition (OR = 1.94, *p* < .01), mental health condition (OR = 2.72, *p* < .05), and any health condition (OR = 2.94, *p* < .01) compared to their heterosexual peers. Compared to White youth, those who identified as Black had lower odds of having a mental health condition, substance use disorder, any condition (*p* < .01), or all three (*p* < .05). Those who identified as Hispanic/Latino had lower odds of substance use (OR = 0.54, *p* < .1) than White youth. Those who identified with other races had greater odds of having a physical health condition (OR = 2.48, *p* < .01) but reduced odds of having mental health (OR = 0.49, *p* < .05), substance use (0.38, *p* < .01), or any health condition (OR = 0.46, *p* < .05). Being employed was associated with significantly reduced odds of having a physical health condition (OR = 0.50, *p* < .05), mental health condition (OR = 0.49, *p* < .01), substance use disorder (OR = 0.58, *p* < .1), tri-morbidity (OR = 0.52, *p* < .1), and any health condition (OR 0.41, *p* < .01).

Adverse life experiences were strongly associated with poorer health. Justice system involvement was associated with greater odds of having a physical health condition (OR = 1.92, *p* < .01), mental health condition (OR = 1.52, *p* < .1), and substance use disorder (OR = 1.89, *p* < .05) and twice the odds of tri-morbidity (OR = 2.23, *p* < .05) and any health condition (OR = 1.97, *p* < .01). Child welfare system involvement was associated with greater odds of having a mental health condition (OR = 1.60, *p* < .05). Receiving public benefits was associated with greater odds of having a physical health condition (OR = 1.99, *p* < .01), mental health condition (OR = 1.61, *p* < .05), and any health condition (OR = 1.80, *p* < .05). Respondents with a history of domestic violence had more than three times higher odds of mental health, physical health, any, or all three conditions (OR = 3.91, 3.77, 3.59, 4.72; *p* > .01).

## Discussion

As hypothesized, the findings from this study indicate that greater duration and frequency of homelessness are associated with poorer self-reported health outcomes among unsheltered young adults in Los Angeles County. Young adults experiencing long-term homelessness had significantly greater odds of having a physical health condition, mental health condition, and substance use disorder as compared to short-term homelessness. Associations of long-term homelessness to poor health outcomes were highly robust to multivariate controls for lifetime experiences, with large and highly significant multivariate associations for physical disability, tri-morbidity, and any health condition. We also observed significant bivariate associations between episodic homelessness and poorer health outcomes, but these associations did not persist after controlling for the sociodemographic and structural differences between trajectory groups. This suggests that episodic clients may be characterized by a unique set of lifetime experiences in cycling between housed, unhoused, and institutional settings that are also associated with poorer health outcomes but does not necessarily indicate that the episodic pattern itself is a cause of deteriorating health [29]. We further note that episodic respondents differed from short-term respondents in the underlying causes of homelessness, being more likely to report mental health issues and prison release as causes of homelessness.

Long-term respondents reported nearly identical reasons for homelessness as short-term respondents. This suggests that short-term and long-term respondents are not fundamentally different in their pathways into homelessness but rather that any differences in health or substance use result from the effects of prolonged exposure to unsheltered homelessness [30]. This finding suggests that our findings for self-reported long-term versus short-term homelessness reflect the cumulative effects of unsheltered homelessness experience in driving morbidity.

While this finding has been observed among sheltered and unsheltered adults, our study reveals the effects of unsheltered homelessness manifesting as early as young adulthood and suggests the possibility that these young adults may experience greater “weathering” due to their increasing health consequences [31–33]. Future research should explore whether other features of weathering, such as an accelerated pattern of aging, are detectable among young adults. Intervention efforts may be most effective if targeted at the primary drivers of homelessness identified in this study: unemployment and household conflict. Eliminating structural barriers to employment and education can help young adults improve financial stability and facilitate economic mobility [34,35]. Reconnecting young people with their families has also been found to promote exits out of homelessness [12,36], although this approach may not be appropriate for LGBTQ who became homeless due to family conflict resulting from their coming out [37].

Our multivariate analysis also aimed to better isolate the impact of homeless trajectory on health by accounting for a rich set of controls capturing the precursors of chronic and episodic homelessness. Experiences of domestic violence and justice system involvement were associated with substantially worse health across all measures. Evidence that receiving public benefits is associated with greater odds of having a physical or mental health condition may reflect that certain conditions are required to qualify for benefits. We note that White youth and male youth were both significantly more likely than other groups to experience episodic or long-term homelessness and to report health conditions. These findings are consistent with a growing body of research that suggests that racial disparities in health may be narrowed or even reversed among highly disadvantaged subgroups due to different mechanisms of selection into homelessness by race/ethnic group. Specifically, homelessness for Black individuals may be driven by economic factors and systemic racism, while homelessness among White individuals may be driven by abuse, neglect, or chronic mental health concerns [38–40]. In keeping with other recent studies of Hispanic homelessness [41], we observed an unusually large Hispanic share in the sample, and these respondents also had significantly lower odds of substance use. Similarly, we find that experiences of domestic violence and system involvement are strongly associated with both poor health outcomes and more prolonged homelessness. Of particular note for this highly overpoliced population, justice system involvement was associated with large and highly significant risks for all adverse health outcomes. Future studies should explore the complex inter-sectionalities between unsheltered homelessness experience and experiences of structural racism, sexism, homophobia, and policing.

This descriptive study carries some limitations. First, all measures were self-reported and not based on validated scales, although all have been widely used in studies of people experiencing homelessness. Second, the measurement of homeless trajectory is limited by the measurement of duration of the current homeless episode rather than total duration of homelessness over a lookback period. Third, the study lacks longitudinal data that could be used to indicate causality. Fourth, the data did not include any measures of social-ecological exposures that could explain the mechanisms of deteriorating health among longer-duration youth. Finally, this study relies on a “point-in-time count” methodology which identifies all individuals experiencing homelessness on a single night or during a specific period of time, which is likely to produce a sample that is not fully representative of the homeless population overall. We further note that due to the dearth of published data exploring the experiences of young people experiencing unsheltered homelessness, and the high rates of unsheltered homelessness in Los Angeles, we focused on this population and did not explore the experiences of those experiencing sheltered homelessness, including couch surfing. This study should serve as an urgent call for longitudinal studies that connect richer and/or objective measures of homeless/housing trajectories to validated measures of health via specific social-ecological exposures and for interventions which target youth homelessness and prevent long-term homelessness among youth.

### Conclusion

This study found that unsheltered youth with longer durations and more frequent episodes of homelessness have substantially poorer health outcomes than those with shorter durations and fewer episodes. The association of longer duration to poorer health outcomes persisted even in the presence of a rich set of controls for demographic/socioeconomic status and a range of adverse life experiences and in spite of the fact that long-term and short-term respondents reported similar reasons for homelessness. Our results thus suggest that longer duration of unsheltered homelessness may drive the onset of mental and physical illness among young people. While earlier studies have associated experiences and duration of homelessness to early onset of conditions among adults, this is the first such evidence that this effect can be observed even in young adulthood (aged 18–24 years). Our results point to the urgent need for prevention and early intervention in young adult homeless trajectories to avert lifelong consequences for economic wellbeing and emerging health disparities.

## Figures and Tables

**Table 1:** Respondent characteristics by homelessness trajectory group

	Short-term	Episodic	Long-term	Total	*P* value
					
	n = 354	n = 283	n = 583	n = 1,220	

Age, mean (SD)	22.3 (2.1)	22.8 (1.6)	22.6 (1.7)	22.6 (1.9)	.019
Gender					.012
Male	59.9%	63.7%	67.6%	64.4%	
Female	37.4%	30.1%	28.7%	31.7%	
Transgender	1.7%	3.7%	3.3%	2.9%	
Gender nonconforming	1.0%	2.5%	0.4%	1.0%	
Sexual orientation					.002
Heterosexual	79.2%	75.8%	78.8%	78.3%	
Gay or lesbian	4.7%	8.9%	9.5%	7.9%	
Bisexual	15.2%	12.3%	9.5%	11.8%	
Unsure/questioning	0.9%	3.0%	2.2%	2.0%	
Race/ethnicity					.631
Hispanic	46.5%	37.8%	38.9%	41.1%	
Non-Hispanic White	16.4%	27.6%	23.1%	21.9%	
Non-Hispanic Black	29.2%	23.4%	28.0%	27.5%	
Other races	7.8%	11.2%	10.1%	9.6%	
Highest level of education completed					.203
Less than high school	36.7%	39.4%	40.3%	39.0%	
High school/GED	47.0%	43.8%	44.5%	45.2%	
Some college or higher	16.3%	16.8%	15.2%	15.9%	
Employment status					.359
Unemployed	80.3%	69.4%	75.7%	75.9%	
Employed	17.9%	27.3%	19.9%	20.7%	
None of the above	1.9%	3.4%	4.4%	3.4%	
Domestic violence					> .001
No	59.1%	42.5%	50.4%	51.6%	
Yes	37.5%	53.6%	43.3%	43.5%	
Not reported	3.4%	3.9%	6.3%	4.9%	
Justice system involvement					> .001
No involvement	53.4%	45.5%	31.9%	41.3%	
Involved	46.6%	54.5%	68.1%	58.7%	
Child welfare system					> .001
No involvement	76.8%	58.5%	63.1%	66.5%	
Involved	23.2%	41.5%	36.9%	33.5%	
Government assistance					.071
Did not receive	37.7%	34.2%	31.1%	33.7%	
Received	62.3%	65.8%	68.9%	66.3%	

Weighted estimates. *p* values for categorical variables based on chi-square tests. Significance for age based on one-way analysis of variance.

**Table 2:** Health conditions and summary health outcomes by homelessness trajectory group

	Means				T-tests (*p* values)	
		
	Short-term	Episodic	Long-term	Total	Episodic versus Short	Long versus Short
						
	n = 354	n = 283	n = 583	n = 1,220		

Health condition						
Physical disability	3.1%	6.3%	8.3%	6.3%	.120	.011
Physical illness	4.2%	6.0%	8.0%	6.4%	.280	.260
Traumatic brain injury	1.6%	3.3%	3.9%	3.1%	.240	.170
Developmental disability	8.2%	4.7%	6.8%	6.8%	.830	.370
Human immunodeficiency virus	1.5%	2.7%	5.7%	3.8%	.090	.150
Serious mental illness	17.4%	26.9%	32.8%	26.8%	.004	> .001
Severe depression	22.4%	33.2%	24.5%	25.5%	.008	.012
PTSD	18.8%	17.3%	26.6%	22.4%	.005	> .001
Problematic alcohol use	10.3%	9.2%	12.4%	11.1%	.110	.040
Problematic drug use	15.3%	17.7%	21.9%	19.0%	.012	> .001
Summary health outcome						
Physical health condition	15.6%	16.5%	26.0%	20.9%	.130	.008
Mental health condition	39.8%	50.5%	53.3%	48.5%	> .001	> .001
Substance use disorder	18.3%	21.3%	25.5%	22.4%	.021	> .001
Tri-morbidity	2.5%	5.4%	7.6%	5.6%	.037	.002
Any heath condition	47.0%	60.0%	69.4%	60.5%	> .001	> .001

Weighted estimates. All *p* values calculated based on pairwise *t*-tests.

**Table 3:** Covariates of health status from multivariate logistic regression models

	Physical health	Mental health	Substance use	Tri-morbidity	Any health condition

Trajectory (ref = short-term)					
Episodic	0.868 (0.229)	1.037 (0.445)	0.963 (0.321)	1.454 (0.569)	1.194 (0.454)
Long-term	1.643 (0.454)*	1.279 (0.431)	1.136 (0.413)	2.139 (0.845)*	1.999 (0.666)**
Age (years)	1.001 (0.0739)	1.081 (0.0711)	0.932 (0.0700)	1.095 (0.0830)	0.991 (0.0617)
Sex/gender (ref = male)					
Female	0.515 (0.143)**	0.674 (0.165)	0.492 (0.148)**	0.865 (0.276)	0.556 (0.107)***
Trans/nonconforming	0.841 (0.292)	0.823 (0.318)	0.914 (0.337)	2.748 (1.047)***	0.511 (0.229)
LGBQ (ref = straight)	1.937 (0.575)**	2.720 (0.927)***	1.610 (0.589)	1.198 (0.367)	2.943 (0.919)***
Race/ethnicity (ref = white)					
Hispanic	1.243 (0.372)	0.652 (0.262)	0.545 (0.180)*	0.687 (0.290)	0.472 (0.182)*
Black/African American	1.539 (0.485)	0.377 (0.123)***	0.319 (0.125)***	0.372 (0.175)**	0.338 (0.107)***
Other race	2.484 (0.771)***	0.490 (0.177)**	0.383 (0.138)***	0.974 (0.437)	0.462 (0.171)**
Experienced Domestic violence (ref = no)	3.912 (1.117)***	3.768 (1.114)***	1.404 (0.425)	3.588 (1.578)***	4.718 (1.416)***
Schooling (Ref = less than HS)					
High school/GED	1.267 (0.299)	0.661 (0.165)*	0.581 (0.155)**	1.161 (0.482)	0.551 (0.123)***
Some college or higher	1.481 (0.353)	0.988 (0.264)	0.869 (0.315)	1.270 (0.469)	0.782 (0.212)
Employment (ref = not employed)					
Employed	0.496 (0.143)**	0.485 (0.111)***	0.586 (0.173)*	0.519 (0.193)*	0.410 (0.0842)***
Employment not stated	0.514 (0.330)	1.871 (0.978)	0.582 (0.240)	0.923 (0.575)	1.596 (0.620)
Justice system involvement (ref = no)	1.922 (0.459)***	1.525 (0.334)*	1.887 (0.495)**	2.227 (0.818)**	1.971 (0.400)***
Child welfare system involvement (ref = no)	0.856 (0.228)	1.603 (0.325)**	1.308 (0.330)	1.376 (0.419)	1.467 (0.305)*
Public benefits recipient (ref = no)	1.985 (0.459)***	1.606 (0.378)**	1.091 (0.337)	0.885 (0.370)	1.803 (0.467)**
Constant	0.033 (0.0511)**	0.074 (0.115)*	1.954 (3.823)	0.002 (0.00312)***	0.987 (1.428)
Observations	1,220	1,220	1,220	1,220	1,220

Weighted estimates.

*** *p* < .01

** *p* < .05

* *p* < .1.
